# Impact of 13-Valent Pneumococcal Conjugate Vaccine on Nasopharyngeal Carriage Rates of *Streptococcus pneumoniae* in a Rural Community in the Dominican Republic

**DOI:** 10.1093/infdis/jiab172

**Published:** 2021-09-01

**Authors:** Maria G Dunn, Fernanda C Lessa, Jacqueline Sánchez, Ramona Cordero, Jesús Feris-Iglesias, Doraliza Cedano, Maria da Glória Carvalho, Josefina Fernández, Kristen A Feemster

**Affiliations:** 1 Department of Pediatrics, Global Health Center and Division of Infectious Diseases, Children’s Hospital of Philadelphia, Philadelphia, Pennsylvania, USA; 2 Division of Bacterial Diseases, Centers for Disease Control and Prevention, Atlanta, Georgia, USA; 3 Hospital Infantil Dr Robert Reid Cabral, Santo Domingo, Dominican Republic; 4 Centro de Salud Divina Providencia, Consuelo, Dominican Republic

**Keywords:** pneumococcal conjugate vaccine, nasopharyngeal colonization, Caribbean, pediatric

## Abstract

**Background:**

Invasive pneumococcal disease (IPD) leads to thousands of pediatric deaths annually. Pneumococcal colonization precedes IPD. In 2013, the Dominican Republic introduced the 13-valent pneumococcal conjugate vaccine (PCV13) into its routine infant immunization program, with doses at ages 2, 4, and 12 months. Prevalence of pneumococcal nasopharyngeal colonization was evaluated post–PCV13 introduction.

**Methods:**

A prospective cohort study of 125 children aged 2–35 months was conducted in a rural Dominican Republic community November 2016 through July 2017. Nasopharyngeal swabs and clinical and vaccination data were collected at enrollment and 4–6 months later. Serotypes included in PCV13 were defined as vaccine-type. Colonization rates and serotype distribution were compared at baseline and follow-up, and the association between colonization and vaccination status among the entire cohort was evaluated at each time point.

**Results:**

Of 125 children enrolled, 118 (94%) completed follow-up. Overall and vaccine-type pneumococcal colonization rates were 62% and 25%, respectively, at baseline and 60% and 28% at follow-up. Among children age-eligible for 3 doses, 50% and 51% were fully vaccinated at baseline and follow-up, respectively. At baseline assessment, children up-to-date for age for PCV13 were less likely to be colonized with vaccine-type pneumococci than children not up-to-date, and the same was found for fully vaccinated children (3 doses) compared to those not fully vaccinated (odds ratios [ORs], 0.38 [95% confidence interval {CI}, .18–.79], and 0.14 [95% CI, .04–.45], respectively). The same associations were not found at follow-up assessment.

**Conclusions:**

Three years post -PCV13 introduction, vaccine-type colonization rates remained high. Low vaccination coverage for 3 PCV13 doses may have contributed. The protective effect of PCV13 on vaccine-type carriage suggests an increase in PCV13 coverage could lead to substantial declines in pneumococcal vaccine-type carriage.

Infections caused by *Streptococcus pneumoniae* (or pneumococcus) are a well-known cause of morbidity and mortality in children [1]. As of 2015, pneumococcal infections were responsible for an estimated 294 000 deaths worldwide in human immunodeficiency virus (HIV)–uninfected children aged <5 years of age [2]. Pneumococcus is found in asymptomatic nasopharyngeal (NP) carriers, and can cause mild disease such as otitis media and sinusitis, as well as more severe or invasive disease including pneumonia, sepsis, and meningitis [3]. Nasopharyngeal carriage of *S. pneumoniae* can precede development of invasive disease, depending on the serotype [4, 5]. More than 90 serotypes of *S. pneumoniae* have been identified; serotypes vary in clinical significance by geographic region, patient age, and other factors [3, 6]. Since the licensing of the heptavalent pneumococcal conjugate vaccine (PCV7) in 2000 by the United States (US) Food and Drug Administration, and subsequent development and introduction of 10-valent and 13-valent pneumococcal conjugate vaccines (PCV10 and PCV13, respectively), numerous countries have noted a reduction in incidence of pneumococcal disease, as well as changes in serotype distribution among both symptomatic patients and asymptomatic carriers [6–8]. Multiple studies have shown that serotype shifts occur after introduction of a new vaccine, often exhibiting replacement of vaccine-associated serotypes with non-vaccine-associated serotypes in the nasopharynx [1, 6, 8, 9]. The clinical consequences of this replacement phenomenon vary greatly by serotype [10]. As such, ongoing surveillance regarding existing serotypes and their epidemiological distribution is vital for future prevention efforts [7].

The Dominican Republic (DR) introduced PCV13 in October 2013 as part of the routine infant immunization program, using a schedule with 2 primary series doses at 2 and 4 months of age and a booster dose at 12 months of age [11]. No catch-up campaign was conducted during introduction. A 2011 study by Feris-Iglesias et al in the DR described pre-PCV13 serotypes in hospitalized children (<18 years of age) with pneumonia and pleural effusion; results indicated that PCV13 would provide >90% coverage of cases if it was introduced [11]. From 2013–2016, at the same hospital in the DR, Tomczyk et al demonstrated PCV13 vaccine effectiveness for the prevention of invasive pneumococcal disease (IPD) of 67.2% among children age-eligible to have received ≥1 dose of PCV13 [12]; however, this study reported low (44%) coverage of the third booster dose of PCV13. A study in the US showed that toddlers are the main transmitters of pneumococci in the community [13], thus raising concerns about potential continued transmission of PCV13 serotypes by undervaccinated children in the DR. The study’s primary aim was to describe overall and vaccine-type pneumococcal NP carriage prevalence and serotype distribution among children in a country with low coverage of the third dose of PCV13. A secondary aim was to evaluate the association between PCV13 vaccination status and vaccine-type pneumococcal carriage, both at 2 individual time points and also in subjects who changed vaccination status between the 2 points.

## MATERIALS AND METHODS

A prospective cohort study was performed among children aged 2–35 months who were current patients at the Niños Primeros en Salud (NPS) program at the Centro de Salud Divina Providencia in Consuelo, DR. Consuelo is a rural municipality in southeastern DR, with a total population of about 30 000, more than 20% of whom are <9 years of age [14]. The NPS program represents a partnership with the Children’s Hospital of Philadelphia and serves children under age 5 years in 7 neighborhoods (*barrios*) in this community. Recruitment, enrollment, and data collection took place between November 2016 and July 2017.

Participants were recruited either at a clinic visit or during a home visit by an NPS doctor or nurse. Home visits are routinely conducted twice weekly as part of the NPS program. Patients with active signs of acute infection (fever) at the time of NP swab collection were excluded, as well as those children with craniofacial abnormalities that would limit NP sample collection. Only children aged 2–35 months in November 2016 were included, because this was the age group eligible for PCV13 vaccine receipt.

After informed consent and eligibility screening, study staff administered an enrollment questionnaire to the caretaker/parent, obtained an NP swab from the participating child, and conducted a chart review to confirm vaccination status and illness history. Charts at NPS included details for vaccines received at NPS, as well as data for those received at other government immunization sites (transcribed from the patient’s government-issued vaccine card). Four to 6 months after enrollment, all study participants received a second visit for completion of a follow-up questionnaire, collection of a second NP swab, and chart review to confirm any additional doses of PCV13 received and illness history since enrollment. Follow-up visits were either conducted on the NPS clinic premises or as a home visit. Both questionnaires included sociodemographic characteristics and clinical data known to be potential factors associated with pneumococcal colonization in prior studies [13, 15, 16]. The characteristics explored were child age, barrio (neighborhood) of residence, breastfeeding status, presence of other children in the home, presence of a wood- or coal-burning stove, smoker in the home, recent history of upper respiratory infection (URI) or hospitalization, recent antibiotic use (including name of antibiotic if known or documented), and history of chronic illness such as asthma. Recent URI was defined as cough or nasal congestion in the last 2 weeks, and recent hospitalization or antibiotic use was defined as occurring within the previous 8 weeks. The baseline questionnaire assessed additional parameters regarding vaccine knowledge and barriers, including caregiver’s previous receipt of vaccination counseling, awareness of the pneumococcal vaccine, experience of vaccine shortage, and barriers to vaccination.

Questionnaires were administered initially on paper and later transferred to a database on the Research Electronic Database Capture (REDCap) system [17].

### Specimen Collection and Processing

Each Copan Diagnostics nylon flocked NP swab was placed in a cryotube containing skim milk-tryptone-glucose-glycerol (STGG) transport medium. Cryotubes were then immediately placed on ice in a portable cooler, vortexed at high speed for 10–20 seconds, and transferred within 6 hours to either a tank filled with liquid nitrogen on the NPS premises, or to a –70°C freezer at Robert Reid Cabral Children’s Hospital (RRCCH) in Santo Domingo, DR. Those samples stored in liquid nitrogen were kept frozen and transported to RRCCH within 2 weeks of collection for transfer to the –70°C freezer, where all samples were ultimately stored until processing.

Initial culture, isolation, and identification of *S. pneumoniae* was performed in the RRCCH microbiology laboratory following previously published methods [18]. For each NP swab in STGG, 200 µL was transferred into 5.0 mL enrichment broth (Todd–Hewitt broth with 0.5% yeast extract) and 1.0 mL of rabbit serum, which was incubated for 5–6 hours at 37°C/carbon dioxide (CO2). After incubation, 10 µL of cultured broth was streaked on 5% sheep blood agar plates and incubated overnight at 37°C in 5% CO_2_. Suspected pneumococcal colonies were tested for optochin susceptibility and bile solubility. All positive isolates, as well as negative swabs, were sent to Boston Medical Center in Boston, Massachusetts, on dry ice via an experienced courier, grouped into 2 shipments for the initial and follow-up samples. Positive isolates of *S. pneumoniae* were serotyped using the Quellung reaction method and serotype-specific sera. These methods were also used to identify individual serotypes when multiple serotypes were present in the same sample. Following published methods, repeat culture using gentamicin-enriched broth was performed on 20 of the negative samples from the initial collection (42% of all negatives) to identify potential false-negative samples [19]. None were positive for *S. pneumoniae*; thus, the remainder of the negative samples were not recultured.

### Statistical Analyses

Descriptive statistics (mean and standard deviation, median and interquartile range, percentage) were used to summarize clinical and demographic variables, colonization prevalence, and vaccination status. Associations between sociodemographic/clinical factors and pneumococcal colonization were evaluated separately at each time point. Univariable logistic regression was used to calculate the odds of colonization by any pneumococcal serotype, and specifically by vaccine serotype (VT), in the presence of each sociodemographic or clinical variable to identify potential confounders.

Variables associated (*P* < .05) with both vaccination status and pneumococcal colonization in bivariate analysis were included in multivariable models. Prior antibiotic use was the only variable that met criteria for multivariable logistic regression.

Measures of vaccination status were up-to-date (UTD) for age, fully vaccinated, and number of vaccine doses. UTD for age was defined as having received at least 1, 2, or 3 doses for children aged ≤4 months, 5–11 months, and ≥12 months, respectively. A child was considered fully vaccinated if they were aged ≥12 months and had received 3 doses of PCV13. The number of valid vaccine doses was defined as the number of vaccine doses received at least 14 days prior to NP sample collection. All analyses were performed using Stata 14 Statistical Software [20].

The study was approved by the institutional review board at the Children’s Hospital of Philadelphia and the Dominican national bioethics committee. This activity was reviewed by the Centers for Disease Control and Prevention (CDC) and was conducted consistent with applicable federal law and CDC policy. Human experimentation guidelines of all institutions were followed. As noted, informed consent was obtained from a parent/guardian of each participant.

## RESULTS

### Study Population

Among 125 enrolled children, 118 completed both study visits. The majority (64%) of children were >12 months old at enrollment with a median age of 17.5 months, and about half (49.6%) were male. The majority of participants self-categorized their race as Dominican or mixed Dominican–Haitian (Table 1).

**Table 1.** Overview of Study Population (N = 125, All Enrolled Children)

Characteristic	No. (%)
Age at enrollment, mo	
≤4	12 (10)
5–11 mo	33 (26)
≥12 mo	80 (64)
Sex	
Male	62 (50)
Race	
Dominican	77 (62)
Haitian	11 (9)
Mixed Dominican–Haitian	33 (26)
Other	4 (3)
Vaccine center typically utilized	
Niños Primeros en Salud clinic, study site	49 (39)
Hospital Angel Ponce, local government vaccine clinic	76 (61)
Vaccine knowledge of parent/guardian	
I have heard of the pneumococcal vaccine	64 (51)
I have been told what vaccines my child needs	57 (46)
Vaccine barriers reported by parent/guardian	
I have taken my child for PCV13 vaccination and been told the center did not have it	43 (34)
I have experienced factors that make it difficult to vaccinate my child	28 (22)
Lack of transportation	5 (4)
Lack of time	6 (5)
Lack of knowledge of when vaccines are due	10 (8)
Unavailability of vaccines at time of visit	3 (2)
Undesirable side effects (fever, pain)	4 (3)
I do not believe vaccines are important	0
PCV13 vaccination status	
Up-to-date for age (all groups) with valid doses^a^	
Sample 1	65 (52)
Sample 2^b^	67 (57)
Up-to-date for age (≤4 mo) with 1 valid dose	
Sample 1 (n = 12)	6 (5)
Sample 2 (n = 0)	0
Up-to-date for age (5–11 mo) with 2 valid doses	
Sample 1 (n = 33)	19 (15)
Sample 2 (n = 23)	19 (16)
Fully vaccinated with 3 doses (age ≥12 mo)	
Sample 1 (n = 80)	40 (50)
Sample 2 (n = 95)	48 (51)
Pneumococcal colonization rates^c^	
Sample 1 (n = 125)	
Any serotype	77 (62)
Vaccine serotype	31 (25)
Sample 2 (n = 118)	
Any serotype	71 (60)
Vaccine serotype	33 (28)

Abbreviations: PCV13, 13-valent pneumococcal conjugate vaccine.

^a^Doses are considered valid if given at least 14 days prior to nasopharyngeal sample collection.

^b^n = 118 due to loss to follow-up.

^c^Colonization rates calculated by number of subjects colonized, not by number of serotypes isolated.

### Pneumococcal Carriage

Nasopharyngeal swabs were obtained from all enrolled children (n = 125) for sample 1 and all children available for follow-up (n = 118) for sample 2. Overall rates of colonization by any *S. pneumoniae* serotype were 77 of 125 subjects (62%) and 71 of 118 subjects (60%) for samples 1 and 2, respectively, whereas rates for VT were 34 of 125 subjects (27%) and 33 of 118 (28%), respectively. Six subjects in sample 1 and 5 subjects in sample 2 were colonized by multiple serotypes.

**Figure 1.**
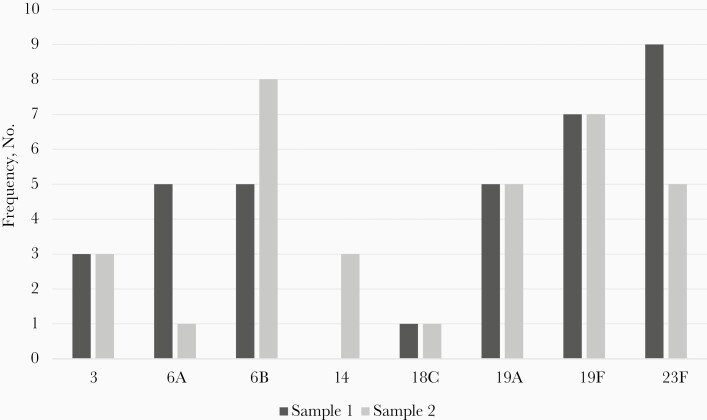
Vaccine-type serotype distribution. *Represents all vaccine-type serotypes isolated. For co-colonized subjects, serotypes were counted individually.

**Figure 2.**
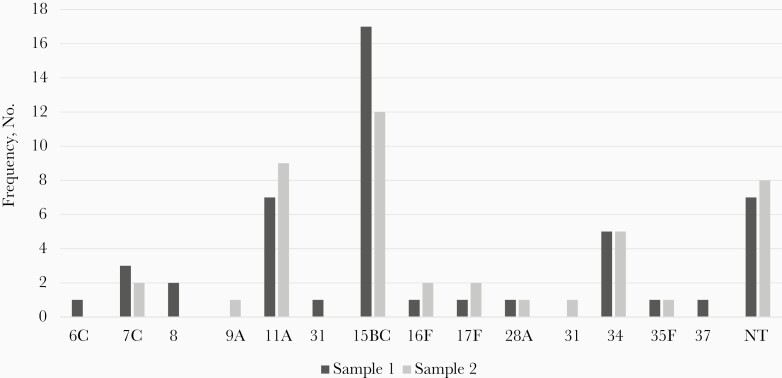
Non-vaccine-type serotype distribution. Abbreviation: NT, nontypeable.

**Figure 3.**
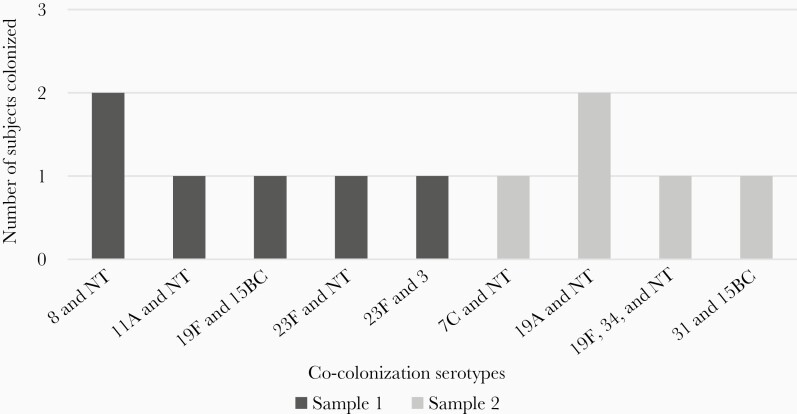
Serotype distribution in co-colonized subjects. Abbreviation: NT, nontypeable.

A total of 83 *S. pneumoniae* colonies were isolated in sample 1 and 77 in sample 2. The most common overall serotype was 15B/C, found in 17 (14%) of the initial and 12 (10%) of follow-up samples. The most common VT serotype differed by sample collection: 23F for sample 1 (n = 9 [7%]) and 6B in sample 2 (n = 8 [7%]) (Figures 1 and 2). Subjects co-colonized with multiple serotypes are shown in Figure 3. Nontypeable (NT) *S. pneumoniae* was the most common co-colonizer; all NT colonies were confirmed to be optochin sensitive and bile soluble.

### Sociodemographic Factors and Association With Colonization

Of the factors included in the questionnaire, only recent antibiotic use was significantly associated with a decreased odds of colonization by any serotype in the first sample (odds ratio [OR], 0.24 [95% confidence interval {CI}, .10–.55]; *P* < .01), but no association was found in the second sample. Subject age, barrio of residence, smoker in the home, wood- or coal-burning stove in the home, breastfeeding status, recent URI or hospitalization, presence of a chronic health condition, and presence of other children in the home were not significantly associated with colonization (see Table 2). At enrollment, 33 caregivers reported their child receiving antibiotics in the prior 8 weeks, with 18 (55%) receiving a penicillin derivative such as amoxicillin and 8 (24%) receiving a cephalosporin; the remaining 7 did not know which antibiotic was used. At follow-up, of the 24 participants who had received antibiotics, 15 (63%) had received amoxicillin, 6 (25%) a cephalosporin, and 3 did not know.

**Table 2.** Demographic and Clinical Characteristics and Colonization Status

Characteristic	No. (%) for Sample 1	No. (%) Colonized by Any Spn Serotype at Sample 1	Association With Colonization by Any Spn, OR (95% CI)	No. (%) Colonized by VT at Sample 1	Association With Colonization by VT, OR (95% CI)	No. (%) for Sample 2	No. (%) Colonized by Any Spn Serotype at Sample 2	Association With Colonization by Any Spn, OR (95% CI)	No. (%) Colonized by VT at Sample 2	Association With Colonization by VT, OR (95% CI)
Total population	125	77 (62%)	…	34 (27%)	…	118	71 (60%)	…	33 (28%)	…
Age of patient										
≤4 mo	12 (10%)	8/12 (67%)	1.28 (.36–4.49), *P* = .71	1/12 (8%)	0.22 (.03–1.78), *P* = .16	0	…	…	…	…
5–11 mo	33 (26%)	21/33 (64%)	1.13 (.49–2.56), *P* = .78	11/33 (33%)	1.50 (.63–3.56), *P* = .34	23 (19%)	17/23 (74%)	2.15 (.78–5.94), *P* = .14	9/23 (39%)	1.90 (.73–4.95), *P* = .19
≥12 mo	80 (64%)	48/80 (60%)	0.83 (.39–1.76), *P* = .62	22/80 (28%)	0.98 (.43–2.24), *P* = .96	95 (81%)	54/95 (57%)	0.46 (.17–1.28), *P* = .13	24/95 (25%)	0.53 (.20–1.37), *P* = .19
Barrio of residence										
Barrio 1	19 (15%)	10/19 (53%)	0.65 (.24–1.73), *P* = .39	4/19 (21%)	0.68 (21–2.20), *P* = .52	18 (15%)	8/18 (44%)	0.47 (.17–1.30), *P* = .14	4/18 (22%)	0.70 (.21–2.30), *P* = .56
Barrio 2	23 (18%)	15/23 (65%)	1.21 (.47–3.11), *P* = .69	9/23 (39%)	1.98 (.76–5.13), *P* = .16	21 (18%)	12/21 (57%)	0.86 (.33–2.23), *P* = .76	5/21 (24%)	0.77 (.26–2.30), *P* = .64
Barrio 3	29 (23%)	19/29 (66%)	1.24 (.52–2.97), *P* = .62	9/29 (31%)	1.28 (.51–3.17), *P* = .60	28 (24%)	20/28 (71%)	1.91 (.76–4.79), *P* = .17	11/28 (39%)	2.00 (.81–4.91), *P* = .13
Barrio 4	37 (30%)	23/37 (62%)	1.03 (.47–2.28), *P* = .93	9/37 (24%)	0.81 (.34–1.96), *P* = .64	34 (29%)	24/34 (71%)	1.89 (.80–4.44), *P* = .14	12/34 (35%)	1.63 (.69–3.86), *P* = .26
Barrio 5	11 (9%)	7/11 (64%)	1.10 (.30–3.98), *P* = .88	3/11 (27%)	1.00 (.25–4.03), *P* = .99	11 (9%)	4/11 (36%)	0.34 (.09–1.24), *P* = .10	1/11 (9%)	0.23 (.03–1.91), *P* = .18
Barrio 6	6 (5%)	3/6 (50%)	0.61 (.12–3.14), *P* = .55	0	…	6 (5%)	3/6 (50%)	0.65 (.12–3.35), *P* = .60	0	…
Smoker in the home										
Yes	18 (14%)	13/18 (72%)	1.75 (.58–5.26), *P* = .31	3/18 (17%)	0.51 (.14–1.90), *P* = .32	18 (15%)	10/18 (56%)	0.80 (.29–2.20), *P* = .67	3/18 (17%)	0.46 (.13–1.73), *P* = .23
No^a^	107 (86%)	64/107 (60%)	…	31/107 (29%)	…	100 (85%)	61/100 (61%)	…	30/100 (30%)	…
Wood or coal-burning stove in the home										
Yes	67 (54%)	39/67 (58%)	0.73 (.35–1.52), *P* = .40	17/67 (25%)	0.89 (.40–1.97), *P* = .78	64 (54%)	37/64 (58%)	0.81 (.38–1.69), *P* = .57	16/64 (25%)	0.73 (.32–1.62), *P* = .44
No	58 (46%)	38/58 (66%)	…	17/58 (29%)	…	54 (46%)	33/54 (61%)	…	17/54 (31%)	…
Breastfeeding status										
Never	14 (11%)	8/14 (57%)	0.81 (.26–2.50), *P* = .71	5/14 (36%)	1.65 (.51–5.32), *P* = .41	14 (12%)	9/14 (64%)	1.22 (.38–3.89), *P* = .74	5/14 (36%)	1.51 (.47–4.89), *P* = .50
Currently	42 (34%)	27/42 (64%)	1.19 (.55–2.56), *P* = .66	11/42 (26%)	0.98 (.42–2.29), *P* = .97	34 (29%)	20/34 (59%)	0.92 (.41–2.08), *P* = .85	7/34 (21%)	0.58 (.22–1.50), *P* = .26
Ever	69 (55%)	42/69 (61%)	1.00 (.60–1.70), *P* = .97	18/69 (26%)	0.81 (.46–1.42), *P* = .46	70 (59%)	42/70 (60%)	0.98 (.46–2.08), *P* = .96	21/70 (30%)	1.29 (.56–2.95), *P* = .55
URI within last 2 wk										
Yes	104 (83%)	63/104 (61%)	0.77 (.29–2.07), *P* = .60	27/104 (26%)	0.67 (.24–1.83), *P* = .43	83 (70%)	54/83 (65%)	1.97 (.88–4.40), *P* = .10	26/83 (31%)	1.82 (.71–4.71), *P* = .20
No	21 (17%)	14/21 (67%)	…	7/21 (33%)	…	35 (30%)	17/35 (49%)	…	7/35 (20%)	…
Hospitalization in last 8 wk										
Yes	8 (6%)	4/8 (50%)	0.60 (.14–2.53), *P* = .49	3/8 (38%)	1.74 (.39–7.72), *P* = .48	4 (3%)	4/4 (100%)	OR, 1	2/4 (50%)	2.68 (.36–19.8), *P* = .34
No	117 (94%)	73/117 (62%)	…	31/117 (26%)	…	114 (97%)	67/114 (59%)	…	31/114 (27%)	…
Antibiotics last 8 wk										
Yes	33 (26%)	12/33 (36%)	0.24 (.10–.55), *P* < .01	7/33 (21%)	0.68 (.26–1.76), *P* = .43	24 (20%)	14/24 (58%)	0.91 (.37–2.26), *P* = .84	7/24 (29%)	1.08 (.40–2.90), *P* = .88
No	92 (74%)	65/92 (71%)	…	27/92 (29%)	…	94 (80%)	57/94 (61%)	…	26/94 (28%)	…
Chronic health condition^b^										
Yes	18 (14%)	8/18 (44%)	0.44 (.16–1.21), *P* = .11	4/18 (22%)	0.77 (.23–2.52), *P* = .66	18 (15%)	11/18 (61%)	1.05 (.37–2.93), *P* = .93	5/18 (28%)	0.99 (.32–3.03), *P* = .99
No	107 (86%)	69/107 (64%)	…	30/107 (28%)	…	100 (85%)	60/100 (60%)	…	28/100 (28%)	…
Other children in the home										
Yes	93 (74%)	57/93 (61%)	0.95 (.41–2.18), *P* = .90	25/93 (27%)	0.89 (.36–2.19), *P* = .80	87 (74%)	53/87 (61%)	1.13 (.49–2.59), *P* = .78	24/87 (28%)	0.93 (.38–2.31), *P* = .88
No	32 (26%)	20/32 (63%)	…	9/32 (28%)	…	31 (26%)	18/31 (58%)	…	9/31 (29%)	…

Abbreviations: CI, confidence interval; OR, odds ratio; Spn, *Streptococcus pneumoniae*; URI, upper respiratory infection; VT, vaccine serotype.

^a^ORs for dichotomous variables are only presented for 1 category per variable.

^b^Chronic health conditions included asthma (9), sickle cell anemia (4), and “others” (5) such as heart defects, adenoid hypertrophy, and global developmental delay.

### Vaccination and Association With Colonization

Half of study participants (52% [65/125]) were UTD for age for PCV13 at initial sample collection, while 57% (67/118) were UTD at follow-up (Tables 1 and 3). Among children UTD for PCV13, overall prevalence of pneumococcal colonization was 51% (33/65) and 58% (39/67) in sample 1 and sample 2, respectively, while prevalence of VT pneumococcal colonization was 18% (12/65) and 27% (18/67), respectively.

**Table 3.** Association Between 13-Valent Pneumococcal Conjugate Vaccine Vaccination Status and Pneumococcal Colonization

Vaccination Status	No. of Doses	Sample 1					Vaccination Status	Sample 2				
		No. (% of Age-Specific Cohort)	Colonized by Any Serotype, No. (%)	Association w Colonization, Any Serotype, OR (95% CI), *P* Value^a^	Colonized by VT, No. (%)	Association With Colonization by VT		No. (% of Age-Specific Cohort)	Colonized by Any Serotype, No. (%)	Association With Colonization by Any Serotype, OR (95% CI), *P* Value^a^	Colonized by VT, No. (%)	Association With Colonization by VT
Unvaccinated (across age groups, compared to UTD for age)	0	13/125 (10%)	11/13 (85%)	5.33, FE *P* = .03	3/13 (23%)	1.33, FE *P* = .71	Unvaccinated (compared to UTD for age)	5/118 (4%)	3/5 (60%)	1.08, FE *P* = 1.00	2/5 (40%)	1.81, FE *P* = .61
UTD, for all groups, compared to not UTD	1, 2, or 3	65/125 (52%)	33/65 (51%)	0.38 (.18–.79), *P* = .01	12/65 (18%)	0.39 (.17–.89), *P* = .02	UTD for age, for all groups	67/118 (57%)	39/67 (58%)	0.83 (.39–1.75), *P* = .62	18/67 (27%)	0.88 (.39–1.98), *P* = .76
Age ≤4 mo (n = 12)							Age ≤4 mo (n = 0)					
UTD for age (compared to not UTD in age group)	1	6/12 (50%)	4/6 (67%)	1.00, FE *P* = 1.00	1/6 (17%)	NC (0 non-UTD subjects VT^+^)	UTD	…	…	…	…	…
Age 5–11 mo (n = 33)							Age 5–11 mo (n = 23)					
Unvaccinated (compared to UTD in age group)	0	5/33 (15%)	5/5 (100%)	NC (0 subjects uncolonized)	2/5 (40%)	1.14, FE *P* = 1.00	Unvaccinated	1/23 (4%)	1/1 (100%)	NC	1/1 (100%)	NC
PV (compared to UTD in age group)	1	9/33 (27%)	7/9 (78%)	3.89, FE *P* = .22	2/9 (22%)	0.49, FE *P* = .67	PV 1 dose	3/23 (13%)	3/3 (100%)	NC	2/3 (67%)	3.71, FE *P* = .53
UTD for age (compared to not UTD in age group)	2	19/33 (58%)	9/19 (47%)	0.15, FE *P* = .03	7/19 (37%)	1.46, FE *P* = .72	UTD	19/23 (83%)	13/19 (68%)	NC (all non-UTD colonized)	6/19 (32%)	0.15, FE *P* = .26
Age ≥12 mo (n = 80)							Age ≥12 mo (n = 95)					
Unvaccinated	0	2/80 (2%)	2/2 (100%)	NC	1/2 (50%)	9.00, FE *P* = .23	Unvaccinated	4/95 (4%)	2/4 (50%)	0.85, FE *P* = 1.00	1/4 (25%)	1.00, FE *P* = 1.00
PV (compared to FV)	1	4/80 (5%)	2/4 (50%)	1.00, FE *P* = 1.00	2/4 (50%)	9.00, FE *P* = .08	PV 1 dose	5/95 (5%)	2/5 (40%)	0.56, FE *P* = 1.00	1/5 (20%)	0.75, FE *P* = 1.00
PV (compared to FV)	2	34/80 (43%)	24/34 (71%)	2.40 (.92–6.29), *P* = .08	15/34 (44%)	7.10, FE *P* < .01	PV 2 doses	38/95 (40%)	24/38 (63%)	1.45 (.61–3.46), *P* = .40	10/38 (26%)	1.07 (.40–2.84), *P* = .89
FV (compared to not FV in age group)	3	40/80 (50%)	20/40 (50%)	0.43 (.17–1.07), *P* = .07	4/40 (10%)	0.14, FE *P* < .01	FV	48/95 (51%)	26/48 (54%)	0.80 (.36–1.81), *P* = .60	12/48 (25%)	0.97 (.39–2.45)*P* = .95

Abbreviations: CI, confidence interval; FE, Fisher exact; FV, fully vaccinated; NC, not calculated; OR, odds ratio; PCV13, 13-valent pneumococcal conjugate vaccine; PV, partially vaccinated; UTD, up to date; VT, vaccine serotype.

^a^For OR *P* values, logistic regression was used if n > 5 in all cells. If n < 5 in 1 or more cells, Fisher exact test was used.

For non-UTD children, overall prevalence of pneumococcal colonization was 73% (44/60) and 63% (32/51) for sample 1 and sample 2, respectively, and VT colonization was 37% (22/60) and 29% (15/51). For sample 1, the odds of colonization by any *S. pneumoniae* serotype were significantly lower in UTD children than in non-UTD children (OR, 0.38 [95% CI, .18–.79]; *P* = .01). The odds of VT colonization were also significantly lower in UTD children (OR, 0.39 [95% CI, .17–.89]; *P* = .02). No associations between PCV13 vaccination status and pneumococcal colonization were observed in sample 2.

For unvaccinated and partially vaccinated subjects stratified by age and number of doses received, prevalence of overall and VT pneumococcal colonization was higher than that for UTD and fully vaccinated subjects at sample 1. In children aged ≥12 months, the odds of colonization were significantly higher for 2-dose partial vaccination than for fully vaccinated subjects (OR, 7.10; Fisher exact *P* < .01). These differences in prevalence, and associations between colonization and vaccination status, were not noted at sample 2, particularly among children aged ≥12 months. These findings are summarized in Table 3.

Among participants ≥12 months of age and eligible to complete the 3-dose series, one-half evaluated at each time point were fully vaccinated (40/80 for sample 1 and 48/95 for sample 2). For these fully vaccinated children, overall pneumococcal colonization for sample 1 and sample 2 was 50% (20/40) and 54% (26/48), respectively, while VT pneumococcal colonization was 10% (4/40) and 25% (12/48). Among subjects ≥12 months and not fully vaccinated with 3 doses, overall pneumococcal colonization for sample 1 and sample 2 was 70% (28/40) and 60% (28/47), respectively, while VT pneumococcal colonization was 45% (18/40) and 26% (12/47).

When examining study subjects who were both fully vaccinated and colonized with VT pneumococci (FV/VT), all 4 children meeting these criteria at sample 1 remained colonized by VT serotypes at sample 2. Of the other 8 children who were categorized as FV/VT at sample 2, 3 had received an interim dose of PCV13 vaccine between samples 1 and 2. At sample 1, fully vaccinated status (3 doses) was significantly associated with a lower odds of VT colonization compared to those who received 2 or fewer doses (OR, 0.14; Fisher exact *P* < .01). There was no significant association between fully vaccinated status and overall or VT colonization at sample 2.

**Figure 4.**
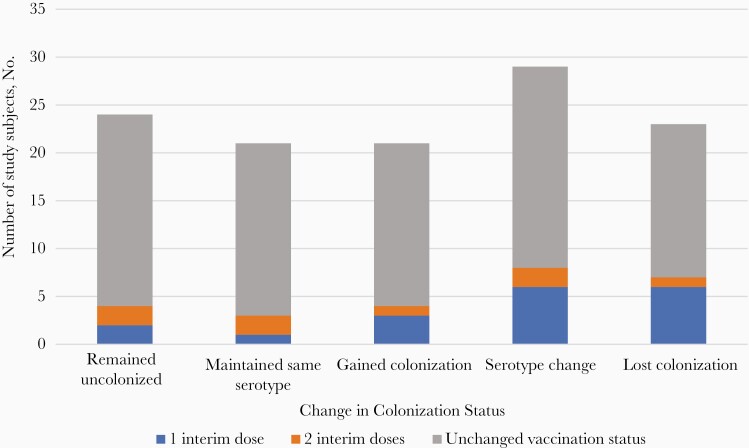
Change in 13-valent pneumococcal conjugate vaccine colonization status according to change in vaccination status.

Of the 118 children who completed both initial and follow-up study visits, the majority (92 [78%]) did not change vaccination status between sample collections. Seventy-three children (62%)—54 without an interim additional dose of vaccine—exhibited a change in pneumococcal carriage status between sample collections, meaning a gain in carriage, loss of carriage, or change in serotype (Figure 4). Due to the small size of the cohort, this study was not adequately powered to measure associations between change in vaccination status and change in carriage status.

### Multivariable Analyses

In adjusted models with antibiotic use as a covariate, there were no significant changes in the association between vaccination status and likelihood of NP carriage for samples 1 and 2 (Table 4). At sample 1, UTD vaccination status remained significantly associated with lower odds of both overall pneumococcal colonization and VT colonization. At sample 2, the lack of association did not change. Similarly, fully vaccinated status at sample 1 remained associated with lower odds of overall colonization, although the point estimate was not statistically significant. Fully vaccinated status did remain significantly associated with lower odds of VT colonization. The lack of association at sample 2 also did not change with adjustment for prior antibiotic use.

**Table 4.** Multivariable Analyses Stratified by Sample Collection Period^a^

Vaccination Status	Colonization by Any Serotype, aOR (95% CI)	Colonization by Vaccine Serotype, aOR (95% CI)
Up-to-date status^b^		
Sample 1	0.39 (.18–.86)	0.36 (.16–.83)
Sample 2	0.82 (.39–1.73)	0.89 (.39–2.00)
Fully vaccinated status^c^		
Sample 1	0.55 (.25–1.24)	0.15 (.04–.54)
Sample 2	0.66 (.31–1.39)	0.78 (.34–1.78)

Abbreviations: aOR, adjusted odds ratio; CI, confidence interval.

^a^Adjusted for antibiotic use.

^b^Up-to-date for age was defined as having received at least 1, 2, or 3 doses for children aged ≤4 months, 5–11 months, or ≥12 months, respectively.

^c^Fully vaccinated status was defined as having received 3 doses if age ≥12 months.

## Discussion

This study among young children in a rural community found high rates (60%) of NP carriage of *S. pneumoniae* at both initial and follow-up sample collections. These rates, even 3 years after PCV13 introduction, were higher than the prevaccine colonization rate of 40% observed in Brazil among children aged 12–23 months, using the same laboratory methods [21].

Community NP carriage rates have not previously been reported in the DR, and this is 1 of only 2 studies to examine pneumococcal serotype distribution after PCV13 introduction [12]. Despite its inclusion in the national immunization program 3 years prior to this study, PCV13 coverage was low in this rural study population, with 52% and 57% UTD for age at initial and follow-up visits, respectively. This is consistent with 2016 findings among an urban pediatric population in Santo Domingo, where 44% of hospitalized children age-eligible to have received ≥1 dose of PCV13 vaccine were UTD for age [12]. Series completion rates were also low: When restricting to children aged ≥12 months, only half of eligible subjects had received all 3 doses at either sample (40/80 [50%] at sample 1 and 48/95 [51%] at sample 2). These low coverage rates stand in contrast to vaccination rates for other routine childhood immunizations in the DR, for example, 84% coverage for diphtheria, tetanus, and pertussis vaccine and 80% coverage for polio [22]. Lower rates of PCV13 vaccination are likely due to its relatively recent introduction (2013) into the extended immunization program compared to other vaccines, and its high implementation costs borne by the DR government, which provides all childhood vaccines [23]. Importantly, low coverage rates may limit the potential population-level impact of vaccination, contributing to the high carriage rates observed in this study.

The most prevalent VTs were 19F, 23F, 6B, and 19A, and the most common nonvaccine serotypes were 15B/C, 11A, and 34. Of the 13 serotypes included in PCV13, no samples were positive for serotype 1, 4, 5, 7F, or 9V. In 1 pre-PCV13 study of IPD in hospitalized Dominican children, the most common serotypes identified were 14, 1, 3, and 6A/6B [11]. Post-PCV13, a case-control study of IPD in the same Dominican hospital most frequently identified serotypes 6A/6B, 14, 3, and 19A [12]. Similar serotype patterns both pre- and post-PCV13 introduction likely reflect the low vaccination coverage of PCV13 in this pediatric community. As this study examined NP colonization in healthy children rather than isolates in subjects with IPD, the higher prevalence of serotypes with less invasive potential (such as 15B/C and 11A) is not surprising [10]. Additionally, serotype 19A carriage has been shown to persist in communities even >5 years after PCV13 introduction, which is consistent with this study’s findings [24].

Being UTD for age at sample 1 was also associated with lower odds of both overall and VT colonization. Receipt of all 3 recommended doses of PCV13 among children ≥12 months was associated with lower odds of VT colonization at sample 1, even after adjusting for antibiotic use in the prior 8 weeks. These associations were not found at sample 2. Partial vaccination did not have statistically significant association with VT colonization status, except for children aged ≥12 months who had received 2 doses at sample 1 (OR, 7.10; Fisher exact *P* < .01).

A higher prevalence of VT colonization was observed from sample 1 to sample 2 for both children UTD with vaccination (18% vs 27%) and fully vaccinated children (10% vs 25%). This was not observed for children who were not UTD (VT prevalence 37% at sample 1 vs 29% at sample 2) or were not fully vaccinated for age (45% vs 26%). The reasons for these differences are unclear. NP colonization in young children is dynamic and highly influenced by their household and community [13]. The majority of children (92 [78%]) did not change vaccination status between samples 1 and 2, so these differences in colonization likely reflect other factors outside of vaccination status, such as colonization prevalence within the subject’s family or surrounding social circle. Recent URI, reported by the majority of subjects (83% and 70% in samples 1 and 2, respectively) may also have influenced colonization rates, as the composition of existing microflora in the upper respiratory tract is known to influence the success of pneumococcal colonization [5].

The role played by the seasonality of pneumococci is also unclear. As observed in other countries, IPD peaks during the rainy season [25]. Sample 2 collection occurred during the months of May and July (months of heavier rainfall), whereas sample 1 collection occurred between November and January (typically drier months). Therefore, seasonality may have played a role in the differences in VT colonization that were observed, independent of vaccination status.

Emerging studies in low- and middle-income countries (LMICs) have documented high NP carriage rates of VT serotypes among vaccinated individuals, even years after PCV13 introduction [26]. All 4 fully vaccinated subjects colonized by VT at sample 1 retained carriage at sample 2. Though 3 of the 12 fully vaccinated subjects with VT carriage at sample 2 had received an interim dose of vaccine, 2 retained carriage by the same VT serotype. Studies in various settings have demonstrated that PCVs have a greater impact on colonization incidence (carriage acquisition) than on carriage clearance [9, 27]. More data are needed from LMICs like the DR, where colonization rates are high, to better understand the drivers of persistent colonization postvaccination.

The overall high rate of colonization by VT serotypes in sample 1 and sample 2 is concerning because it suggests ongoing transmission in the community. Inconsistent or heterogeneous vaccination can create “pockets of persistence” in pneumococcal colonization, similar to this study’s findings [13].

Multiple studies have shown that a booster dose given after 12 months of age is important for overall immunity and may actually be the most important dose of the pneumococcal vaccine series [28]. In the study population, the majority of subjects were >12 months of age (64% and 81% at samples 1 and 2, respectively) and therefore eligible for 3 doses, but half were missing the third dose of vaccine. Considering the clinical importance of the third dose on immunity, it is important to understand barriers to immunization and reasons for noncompletion of the series. One-third of mothers reported facing a lack of vaccine supply when they took their children to be immunized.

This study had several limitations. Specimens had to be transported not only from the rural site of collection to the capital city, but also from the DR to the US. Some pneumococcal colonies could have been missed in this process. Attempts to mitigate this risk were made by following laboratory standard protocols established by the CDC, as well as by reculturing a proportion of negative samples (20 samples [42%]) to assess for missed positives. All remained negative, indicating solid methodology and consistency across both laboratory sites.

The study was also limited by its rural setting, thus limiting generalizability. Risk factors may be different in this cohort compared with the rest of the Dominican population. More information is needed on serotype distribution across different community types in the DR. However, the low PCV13 coverage found in this study is similar to what was found in Santo Domingo and also similar to the World Health Organization/United Nations Children’s Fund coverage estimates [22].

Last, the study population was small and at times was not sufficiently powered to show statistically significant associations between clinical or sociodemographic factors and colonization. The small cohort size, coupled with existing low vaccination coverage in this population, may account for the inconsistent association between colonization and vaccination status noted from sample 1 to sample 2. This was intended to be a pilot study to provide baseline data for future larger-scale studies.

Future studies could inform continued vaccination efforts and monitor pneumococcal serotype distribution. Responding to the need for greater access to pneumococcal vaccines, the DR immunization program (Programa Ampliado de Inmunización) has taken steps to begin a catch-up campaign. This will provide all children 1–4 years of age with a booster dose of PCV13, regardless of previous vaccination status. Based on these findings, this campaign has the potential for great impact on VT pneumococcal NP carriage in Dominican children.

## Supplementary Data

Supplementary materials are available at *The Journal of Infectious Diseases* online. Consisting of data provided by the authors to benefit the reader, the posted materials are not copyedited and are the sole responsibility of the authors, so questions or comments should be addressed to the corresponding author.

## Notes

Presented in part: 11th International Symposium on Pneumococci and Pneumococcal Diseases, Melbourne, Australia, April 2018. Abstract 0504.

***Acknowledgments.*** The authors express their appreciation of the children and families who agreed to participate in this study. They are grateful for all the laboratory expertise and support provided by Boston Medical Center, especially Brent Little and Dr Sabine Schnyder. They acknowledge the project assistance provided by the staff at Niños Primeros en Salud, in particular by Dr Ingrid Japa, Mioced Ramírez, and Heriberto Díaz. They are also thankful for the support of the Children’s Hospital of Philadelphia Global Health Center faculty and staff, including Dr Andrew Steenhoff, Dr Elizabeth Lowenthal, and Adriana Deverlis.

***Supplement sponsorship.*** This supplement is sponsored by the World Health Organization and the U. S. Centers for Disease Control and Prevention.

***Financial support.*** This work was supported by the Global Health Center, Children’s Hospital of Philadelphia (Global Health Pilot Grant to M. D.). In-kind support was provided by Robert Reid Cabral Children’s Hospital and the CDC.

***Potential conflicts of interest.*** All authors: No reported conflicts of interest.
